# Cognitive and emotional factors related to COVID-19 among high-risk ethnically diverse adults at the onset of the New York City outbreak: A cross-sectional survey

**DOI:** 10.1057/s41599-023-01679-x

**Published:** 2023-05-17

**Authors:** Rita Kukafka, Mari Millery, Samuel Pan, Thomas B. Silverman, Tianmai Zhang, Julia E. McGuinness, Katherine D. Crew, Alejandra N. Aguirre

**Affiliations:** 1 Department of Biomedical Informatics, Vagelos College of Physicians and Surgeons, Columbia University Irving Medical Center, New York, NY USA; 2 Herbert Irving Comprehensive Cancer Center, Columbia University Irving Medical Center, New York, NY USA; 3 Department of Sociomedical Sciences, Mailman School of Public Health, Columbia University Irving Medical Center, New York, NY USA; 4 Department of Medicine, Vagelos College of Physicians and Surgeons, Columbia University Irving Medical Center, New York, NY USA; 5 Department of Epidemiology, Columbia University Irving Medical Center, New York, NY USA; 6 Irving Institute for Clinical and Translational Research, Columbia University Irving Medical Center, New York, NY USA

**Keywords:** Health humanities, Psychology

## Abstract

A cross-sectional survey was conducted among high-risk, racially/ethnically diverse adults at the point in time when New York City (NYC) became the COVID-19 pandemic’s global epicenter. The study objective was to assess the threat and coping appraisals (cognitive factors known to correspond with people’s willingness to adopt behaviorally focused interventions) and levels of distress, anxiety, and intolerance for uncertainty (emotional factors). Survey respondents were recruited in April 2020 using an online survey with unpaid recruitment on the GetHealthyHeights.org community-oriented website. We also recruited participants that engaged in previous research studies to gain survey responses from community members at higher risk for COVID-19 complications due to comorbidities compared to the general population. Analysis was performed to test for differences in survey responses by comorbidities, age, race, ethnicity, and employment status. Results show that the devastating effects of the pandemic appear to have uniquely impacted minority respondents, who reported significantly higher levels of anxiety and were significantly more likely to report having little control over whether they will get COVID-19 compared with White/non-Hispanic respondents. Minority respondents also had significantly higher mean scores on the behaviorally focused dimension of the intolerance of uncertainty (IU) scale, which measures avoidance and paralysis in the face of uncertainty. In multivariate analysis, IU predicted anxiety levels, and this association was not mediated by cognitive factors (threat and coping appraisals). By conducting this survey early in the pandemic, our study uniquely evaluated cognitive and emotional factors among a racially/ethnically diverse group of NYC residents during the height of the COVID-19 pandemic. Our findings suggest the need to acknowledge the disparities that appear to exist in pandemic response and for culturally tailored messaging and interventions. Few studies have reported differences by race and ethnicity during pandemic exposure. Therefore, further research on factors that may influence pandemic response among minority populations is needed.

## Introduction

Beginning in late 2019, the novel coronavirus disease 2019 (COVID-19), began to rapidly spread across the globe, becoming an unprecedented public health crisis. While the COVID-19 impact was global, New York City (NYC) quickly became one of the pandemic’s global epicenters. By mid-March, infection rates were five times higher than in the rest of the U.S., with cases one-third of total confirmed cases in the country (Centers for Disease Control and Prevention). In an effort to contain the spread of COVID-19, the state governor’s office implemented restrictions on businesses, schools, social gatherings, and the use of public transportation. These restrictions were fundamentally the same behaviorally focused nonpharmaceutical interventions (NPIs) implemented during the 1918 pandemic, including social distancing and school closures (Morse, 2007; Markel et al., 2007), but unprecedented for the present-day ethnic and diverse populations living and working in the NYC area.

In the absence of pharmaceutical interventions, human behavior is central to curtailing the transmission of COVID-19, which is why the implementation of behaviorally focused NPIs in NYC was the first critical step to controlling the infectious aspect of the pandemic. At the March 31, 2020, White House Coronavirus Task Force Briefing, Dr. Debra Birx reinforced the importance of NPIs, stating, “there is no magic bullet, no magic vaccine or therapy. It’s just behaviors.” The journey that began in March in the early months of the pandemic in NYC was characterized by high mortality, high population-level fear, and extreme anxiety (Thompson et al., 2020; Weinberger-Litman et al., 2021). Living in at-home lockdown, homemade masks, and do-it-yourself haircuts was a new reality for most of the city’s population. Complaints to 311, the city’s hotline to access non-emergency services, rose significantly in telling categories. For example, there were 16,901 calls in a brand-new category, lax social distancing (Wilson, 2020). In NYC, 311 served to understand how the population reacted to COVID-19 in real-time and helped to understand how people respond to NPIs, such as the social-distancing policy, which is vital in reducing COVID-19 infections (Lieberman-Cribbin et al., 2020).

Early studies examining NYC’s COVID-19 response mainly focused on understanding the disease, its epidemiology, and treatment (Ogedegbe et al., 2020; Roy and Ghosh, 2020; DiMaggio et al., 2020; Benitez et al., 2020). Yet, understanding antecedents of health behaviors is a key step to developing effective interventions to increase adherence to NPIs and vaccines once they become available (Liu et al., 2020; Bavel et al., 2020). Literature amassed during previous pandemics incorporated socio-cognitive models to examine theoretically informed antecedents of health behavior to motivate behavioral change, such as knowledge and risk perception. While most constructs within these models are cognitive, emotional constructs can also explain the drivers of health behaviors during a pandemic. For example, the right amount of anxiety about a health threat helps make recommended behaviors more likely, but too much anxiety results in either unnecessary or defensive avoidance behaviors (Rubin et al., 2010). From a different disciplinary lens, a clinical psychology perspective, identifying emotional factors suggests mechanisms to link exaggerated emotional responses to improve favorable outcomes with implications for developing psychotherapeutic interventions (Coelho et al., 2020; Voitsidis et al., 2020). Rarely attempts have been made to layer cognitive-behavioral constructs and emotional factors to inform a comprehensive multi-faceted intervention that increases adherence to NPIs while supporting and maintaining psychological well-being. This crosswalk is critical given the importance of health behaviors within the emotionally laden context of a pandemic.

### Cognitive antecedents of behavior and emotional factors in response to infectious disease outbreaks

Research amassed from previous infectious disease outbreaks establishes key factors related to characterizing cognitive antecedents of behavior which are vital during the spread of pandemic disease and have been valuable in predicting how people respond to emerging infectious diseases (Bavel et al., 2020; Lee and You, 2020; Shinan-Altman and Levkovich, 2020; Vally, 2020; Bish and Michie, 2010; Smith, 2006; Leppin and Aro, 2009). The Protection Motivation Theory (PMT), applied in studies of SARS and HINI (Rogers, 1975; Weston et al., 2020) identified two general and seminal antecedents of behavior labeled threat and coping appraisals, which we also examined in this study. A threat appraisal involves a consideration of the severity of the health threat and a perception of personal vulnerability to it (i.e., perceived risk). Coping appraisal involves whether the health action is perceived to be an effective means of alleviating the threat (i.e., response efficacy) and a consideration by the individual of whether they will be able to carry out the health action (i.e., their perceived self-efficacy). Collectively threat and coping appraisals have explained the motivation for how people behave in past pandemics (Bish and Michie, 2010; Teasdale et al., 2012; Taha et al., 2014; Hornik et al., 2021), highlighting the influence of cognitive foundations for adopting behaviorally focused NPIs to reduce the spread of disease during outbreaks.

There is also evidence that emotional factors, including heightened anxiety levels, are associated with a greater chance of carrying out protective behaviors and therefore are an essential driver of behavior during a pandemic (Taylor and Asmundson, 2020). Prior research found that coping appraisals combined with anxiety related to the health threat can lead to inappropriate, discriminatory actions, for example, avoidance of individuals of Asian descent in response to the 2003 SARS outbreak (Puterman et al., 2009) and more recently during the COVID pandemic (Cho et al., 2020). Studies emphasize the need to support people with vulnerability factors, such as heightened anxiety during pandemics, as they seem prone to heightened distress and intensified behavioral responses (Sauer et al., 2020).

Previous studies relying on explanatory variables from behavior theories to predict how people respond to emerging infectious diseases have seldom focused on intolerance of uncertainty (IU). IU is defined as the “individual’s dispositional incapacity to endure the aversive response triggered by the perceived absence of salient, key, or sufficient information, and sustained by the associated perception of uncertainty.“ (Carleton, 2016; Satici et al., 2020) IU has been associated with increased anxiety and depression and therefore has a vital role in illuminating how people might respond to and cope during a pandemic (Voitsidis et al., 2020; Taha et al., 2014; Carleton, 2016; Einstein, 2014). The level of uncertainty that can be tolerated is a trait individuals bring into an ambiguous situation, which predicts how they might appraise uncertain events. Because high IU has been found to exacerbate the relationship between daily stressors and increased anxiety (Voitsidis et al., 2020; Bakioglu et al., 2020), changing negative beliefs about uncertainty and improving coping strategies have been suggested as practical approaches to influence IU during pandemics (Gu et al., 2020).

### Study objective

In April 2020, we conducted a historically time-sensitive cross-sectional survey among high-risk ethnically diverse adults in NYC. The study objective was to determine their threat and coping appraisals (cognitive factors known to correspond with people’s willingness to adopt behaviorally focused NPIs) and their levels of distress, anxiety, and tolerance for uncertainty (emotional factors). To understand the impact of the pandemic on population sub-groups, we assessed socio-demographic factors on each of these categories of variables and on levels of knowledge, healthcare utilization, and initiation of preventive behaviors. As IU is known to have pronounced psychological ramifications during pandemics, we further investigated the predictive and moderating effects of cognitive threat and coping appraisals, IU, and anxiety.

## Methods

### Design and setting

A cross-sectional design was used, incorporating an online survey with unpaid recruitment on the GetHealthyHeights.org community-oriented health information website. GetHealthyHeights was designed using community-based participatory research for the medically underserved, urban, predominantly Latino community of Washington Heights-Inwood (WAHI) (Millery et al., 2015, 2017). A pop-up on the website was added on April 9, 2020, inviting respondents to share how the outbreak has affected their health and well-being. In addition, emails inviting respondents to complete the survey on the website were sent to participants engaged in three community-based research studies on breast cancer prevention and the Database Shared Resource at Columbia University Irving Medical Center (CUIMC). Participants accessed the survey through a link provided in the invitation email, and a link to the survey was provided through GetHealthyHeights. The first page of the survey was to consent participants. It described the research project, and participants were asked to agree to participate before proceeding to the survey. This solicitation to CUIMC research participants was conducted to gain survey responses from community members at higher risk for COVID-19 complications due to comorbidities compared to the general population. The CUIMC Institutional Review Board approved the study procedures. Data were collected from April through July 2020.

## Questionnaire design

### Sociocultural, healthcare utilization, and demographic factors

We selected questions from the validated Flu Telephone Survey Template (FluTEST), which was designed to assess perceptions and behavior during influenza-like pandemics (Rubin et.al., 2014). FluTEST includes core sets of outcome variables relating to the presence of flu-like illness and to various protective behaviors, as well as a set of likely predictor variables for the behaviors. Evidence-based and evaluated for reliability and non-response bias, the extensive set of items compiled was designed in consideration of the rapid need for survey items as soon as the next pandemic arises. Information collected on demographics and background included age, sex, gender, racial/ethnic background, employment, and current medical conditions that could increase the risk for susceptibility to and/or complications from COVID-19. In addition, we asked whether participants had been tested, were told by a healthcare provider, or believed they had been infected with COVID-19, whether access to healthcare was interrupted, and whether they lost a job due to the pandemic.

### Compliance with behavioral recommendations

Eight items assessed change in health behaviors over the past 4 days because of COVID-19, e.g., Increasing the amount I clean or disinfect, putting a face mask on before going outside. The eight items were rated on a four-point scale (yes, no, not sure, not applicable).

### Cognitive Factors

Eight items assessed knowledge of coronavirus. COVID-19 threat and coping appraisals were assessed based on the PMT (Rogers, 1975). The construct of perceived susceptibility was assessed by two items, i.e., “My chances of getting infected with Coronavirus COVID-19 are greater than other people”. Perceived severity was assessed by three items, i.e., “COVID-19 would be a serious illness for me”. Perceived self-efficacy was assessed by “I have little control over whether I will get COVID-19.” Response efficacy was evaluated by “If I don’t take any preventive actions, then I am likely to get Coronavirus COVID-19.” The items were rated on a five-point scale ranging from 1 (strongly disagree) to 5 (strongly agree).

### Emotional factors

Intolerance of uncertainty (IU) was assessed using the IUS-12 (Carleton et al., 2007), comprised of 12 items to assess reactions to impending uncertainty, ambiguous situations, and the future. It consists of two factors. Prospective anxiety measures a desire for predictability, preference for knowing what the future holds, anxiety about future uncertain events, and active engagement in seeking information to increase certainty. This factor consists of 7 items, e.g., “I can’t stand being taken by surprise”. The second factor, inhibitory anxiety, is the behaviorally focused dimension of IU that measures avoidance and paralysis in the face of uncertainty. It consists of 5 items, e.g., “I must get away from all uncertain situations”. Each item of the IUS-12 is assessed using a 5-point Likert scale ranging from 1 (not at all characteristic of me) to 5 (entirely characteristic of me). There is evidence for the reliability and construct validity of the IUS-12 within non-clinical and community samples. Significant support was found for composite reliability of the total IUS–12 scores (*ρ* = 0.92, 95% CI [0.91, 0.93]) and the inhibitory (*ρ* = 0.87, 95% CI [0.85, 0.89]) and prospective (*ρ* = 0.87, 95% CI = [0.86, 0.89]) subscale scores.

To assess anxiety, we administered a short form of the state scale of the Spielberger State-Trait Anxiety Inventory (STAI-6) (Marteau and Bekker, 1992), adapted from previous studies for use in this study. The 6-item state version of the State-Trait Anxiety Inventory (STAI-6), which assesses anxiety symptoms, was adapted from previous studies for use in this study. The participants reported their current general level of anxiety according to six statements (e.g., “I am worried”) on a 4-point Likert scale (“not at all” = 1, to “very much” = 4). The STAI-6 has been shown to be highly correlated with the 20-item STAI, and all internal consistency reliabilities are greater than 0.90 (Tluczek et al., 2009). The STAI-6 has been used both as self-administered and online questionnaires.

We used the Impact of Event scale revised (IES-R) to measure distress. IES-R consists of 22 items in three subscales: (a) the intrusion subscale with eight items related to intrusive thoughts, nightmares, intrusive feelings and imagery, and dissociative-like re-experiencing, and (b) the avoidance subscale with eight items related to feelings, situations, and ideas and (c) the hyperarousal subscale with six items related to anger, irritability, difficulty concentrating, hypervigilance and heightened startle. Each item is rated by the participants on a scale from 0 to 4 (0 = “not at all,” 1 = “a little bit,” 2 = “moderately,” 3 = “quite a bit,” and 4 = “extremely”) for the past seven days. Importantly, compared to other self-reported measures of psychological impact, the advantage of using the IES is that the event can be specified. The IES-R adapted for COVID-19 has been found to be a valid measure of traumatic stress symptoms associated with the COVID-19 pandemic (Aljaberi et al., 2022; Zhang et al., 2021). The IES-R with modifications for COVID-19 showed good internal validity (*α* = 0.96) (Gottlieb and Schmitt, 2022). We adjusted the IES-R for the case of the COVID-19 pandemic, asking the participants to indicate how distressing each difficulty has been for them during the past seven days with respect to the COVID-19 pandemic.

## Statistical methods

Univariate analysis was performed using Chi-square tests (and Fisher’s exact tests where appropriate), and Student *t*-tests (or nonparametric alternatives such as the Wilcoxon rank-sum test where appropriate) to test for any differences in survey responses by comorbidities, age, race, ethnicity, and employment status. Multivariate analysis was performed using logistic regression to assess potential factors impacting perceived susceptibility, seriousness, and control. Odds ratios (OR) and 95% Confidence Intervals (CI) were reported. A *p*-value of <0.05 was considered to indicate statistical significance. Data analyses were performed using SAS 9.4 (SAS Institute). When determining the sample and its size, we considered the following factors: (a) participants should be from NYC and mainly Washington Heights in upper Manhattan since GetHealthyHeights.org is focused on this largely underserved minority population; (b) data collection in this time-sensitive historical survey was limited given the goal of assessing the short-term impact of COVID-19; and (c) budget, given this was an unpaid sample.

To calculate power from our data, we observed a proportion of 0.14 non-Hispanic Whites reported feeling the loss of control, with a proportion of 0.32 for all other ethnic groups. Based on these proportions, we have an effect size of 0.45. Using our sample size of 182 non-Hispanic Whites and 90 other ethnic groups, we have a power of >96% using a 1-sided proportional *Z*-test at 0.05 alpha. We repeated the power analysis adjusting for four additional outcomes. Using our sample size of 182 non-Hispanic Whites and 90 other ethnic groups, we have a power of >89% using a 1-sided proportional *Z*-test at 0.05 alpha, with adjustment for four outcomes (perceived severity, perceived risk, response efficacy, self-efficacy).

## Results

### Sample characteristics

Table 1 summarizes respondent characteristics. The mean age of respondents was 62 years (range, 25–87 years), 25% identified as Hispanic or Latino, and 82% were female. Thirty-seven (13.5%) were not working, and 36% were retired. All had at least one chronic condition, with 29% reporting having cancer, 18% reporting mental illness, and 17% breathing disorders. Given that COVID-19 testing was mostly unavailable early in the pandemic, only 10% reported that they were tested.

**Table 1:** Sample characteristics (*N* = 321).

	*M* (range) or *N* (%)
Age (years)	62 (25–87)
*Gender*	
Male	51 (18%)
Female	225 (82%)
*Race*^a^	
Asian	8 (3%)
Black or African American	22 (9%)
Native American or Alaska Native	2 (1%)
White	207 (85%)
Multiple	6 (2%)
*Ethnicity*^a^	
Hispanic or Latino	68 (25%)
Not Hispanic or Latino	202 (75%)
*Geographic area*	
NYC or surrounding commutable areas	246 (91%)
Other regions of the U.S.	24 (9%)
*Employment*	
Working full time	102 (37%)
Working part-time	37 (13.5%)
Not working	37 (13.5%)
Retired	99 (36%)
*Lost job/had hours reduced due to COVID-19*	
Yes	42 (16%)
No	152 (55%)
*Respondent believes s/he was Infected with COVID-19*	
Yes	43 (15%)
No	174 (58%)
Don’t Know	81 (27%)
*Tested for COVID-19*	
Yes	30 (10%)
No	266 (90%)
*Respondent told by health care provider that s/he likely has COVID-19*	
Yes	22 (7%)
No	273 (92%)
*Chronic conditions*	
Breathing disorders	56 (17%)
Cancer	94 (29%)
Diabetes	21 (7%)
Kidney Disease	11 (3%)
Mental Illness	58 (18%)
Reported as “other”, not specified	90 (28%)

^a^To explore the impact of minority status in subsequent analyses, race and ethnicity were categorized into two categories: White/non-Hispanic and Minority, which included Hispanic and non-Hispanic Black, Asian (*N* = 2), and respondents reporting multiple races (*N* = 4).

### Self-reported behaviors and healthcare utilization

With respect to behavior, all respondents reported changing at least one health behavior over the past 4 days due to COVID-19, including wearing a mask (94%), washing hands more frequently (94%), staying home unless doing something absolutely essential (90%), cleaning frequently (85%), and using a hand sanitizer (75%) (Table 2). Results showed that White/non-Hispanic respondents were significantly more likely to report cleaning more frequently (<80% vs. <90%, *p* = 0.02) and to discuss a plan if they do contract COVID-19 (72% vs, 28%, *p* = 0.006), compared with minority respondents. Regarding healthcare utilization, 51% reported not being able to receive care through their provider as planned, and only 17% reported receiving healthcare through telemedicine. These data on enacting protective behaviors and disruptions in healthcare utilization demonstrate that most respondents reported adopting key behavioral NPIs, while at the same time, routine healthcare was disrupted with low utilization of healthcare through telemedicine.

**Table 2:** Self-reported behaviors and healthcare utilization.

*“Over the past four days, I have…. because of COVID 19”*	
Item	*N* (%)
Put on a face mask before going outside	268 (94%)
Hand my hands with soap and water more often	268 (94%)
Deliberately canceled or postponed an event	175 (61%)
Increased the amount I clean/disinfect	234 (85%)
Followed a healthy diet or took vitamin supplement	231 (81%)
Carried sanitizing hand gel when out and about	215 (76%)
Stayed home unless I was doing something essential	258 (90%)
Discuss with friends/family what we would do if one of us catches COVID-19	165 (58%)
*Healthcare utilization*	
Able to receive healthcare through telemedicine	44 (17%)
Not able to receive healthcare through provider	142 (51%)
Missed mammogram	44 (17%)
Missed other appointment	156 (56%)

### Cognitive factors: knowledge, perceptions of threat, and coping appraisals

Overall, knowledge about COVID was high, with a mean score of respondents correctly answering 7.27 of the eight questions (SD = 1). Eighty-three respondents (*N* = 242) correctly answered at least seven of the eight knowledge questions.

Table 3 shows the association between respondent characteristics and threat and coping appraisals. The results show that minority respondents rated their likelihood of getting COVID-19 if they didn’t take preventive actions significantly higher (92% vs. 83%, *p* = 0.04) compared to white/non-Hispanic respondents. This same group reported having little control over whether they will get COVID-19 (32% vs. 14%, *p* = 0.0003), but compared with white/non-Hispanic respondents were not more likely to agree that “COVID-19 would be a serious illness for me” (66% vs. 57%, *p* < 0.17). With respect to comorbidities, respondents reporting cancer, diabetes, breathing disorders, and other chronic conditions were significantly more likely to agree that “COVID-19 would be a serious illness for me” compared with respondents not reporting these conditions (68% vs. 55%, 95 vs. 56%, 85% vs. 53%, 74 vs. 52%), respectively. Age was also a factor, with older respondents aged 65 agreeing that “COVID-19 would be a serious illness for me” compared with younger respondents (*p* = 0.0001), while younger respondents aged 18–45 reported having little control over whether they will get COVID-19 (*p* = 0.0003). Non-working respondents were significantly more likely to report that “COVID-19 would be a serious illness for me” compared with working respondents (74% vs. 53%, *p* < 0.001). These results reveal significant variability in respondents’ perceptions and threat and coping appraisals, particularly with respect to age and minority status.

**Table 3:** Threat and coping appraisals across sample characteristics.

	Perceived Risk			Perceived Severity			Response Efficacy			Self-Efficacy		
	My chances of getting infected with Coronavirus COVID-19 are greater than other people.			COVID-19 would be a serious illness for me.			If I don’t take any preventive actions, then I am likely to get Coronavirus COVID-19.			I have little control over whether I will get COVID-19.		
	Yes	No/unsure	*p-*value	Yes	No/unsure	*p*-value	Yes	No/Unsure	*p-* value	Yes	No/Unsure	*p*-value
*Race*												
White, non-Hispanic	63 (34.6%)	119 (65.4%)	0.12	104 (57.1%)	78 (42.9%)	0.17	151 (83.0%)	31 (17.0%)	**0.04**	25 (13.5%)	157 (86.3%)	**0.0003**
Minority	40 (44.4%)	50 (55.6%)		58 (65.9%)	30 (35.1%)		83 (92.2%)	7 (7.8%)		29 (32.2%)	61 (67.8%)	
*Age*												
18–45	23 (36.5%)	40 (63.5%)	0.33	26 (41.9%)	36 (58.1%)	**<0.0001**	53 (84.1%)	10 (15.9%)	0.37	26 (41.3%)	37 (58.7%)	**<0.0001**
46–64	35 (33.3%)	70 (66.7%)		52 (50.0%)	52 (50.0%)		94 (89.5%)	11 (10.5%)		17 (16.2%)	88 (83.8%)	
65+	51 (42.9%)	68 (57.1%)		91 (76.5%)	28 (23.5%)		99 (83.2%)	20 (16.8%)		19 (16.0%)	100 (84.0%)	
*Gender*												
Male	24 (47.1%)	27 (52.9%)	0.11	38 (74.5%)	13 (25.5%)	**0.02**	40 (78.4%)	11 (21.6%)	0.09	10 (19.6%)	41 (80.4%)	0.96
Female	79 (35.1%)	146 (64.9%)		126 (56.5%)	97 (43.5%)		197 (87.6%)	28 (12.4%)		45 (20.0%)	180 (80.0%)	
*Employed*												
Yes	48 (34.5%)	91 (65.5%)	0.31	64 (46.7%)	73 (53.3%)	**<0.0001**	118 (84.9%)	21 (15.1%)	0.66	30 (21.6%)	109 (78.4%)	0.51
No	55 (40.4%)	81 (59.6%)		100 (73.5%)	36 (26.5%)		118 (86.8%)	18 (13.2%)		25 (18.4%)	111 (81.6%)	
*Lost Job*												
Yes	10 (23.3%)	33 (76.7%)	0.06	18 (41.9%)	25 (58.1%)	0.09	37 (86.1%)	6 (14.0%)	0.90	4 (9.3%)	39 (90.7%)	0.08
No	59 (38.8%)	93 (61.2%)		85 (56.7%)	65 (43.3%)		132 (86.8%)	20 (13.2%)		33 (21.7%)	119 (78.3%)	
*Cancer*												
Yes	41 (45.6%)	49 (54.4%)	0.07	61 (67.8%)	29 (32.2%)	**0.05**	72 (80.0%)	18 (20.0%)	0.06	17 (18.9%)	23 (81.1%)	0.45
No	68 (34.5%)	129 (65.5%)		108 (55.4%)	87 (44.6%)		174 (88.3%)	23 (11.68%)		45 (22.8%)	115 (77.2%)	
*Diabetes*												
Yes	15 (71.4%)	6 (28.6%)	**0.001**	20 (95.2%)	1 (4.8%)	**0.0003**	16 (76.2%)	5 (23.8%)	0.20	5 (23.8%)	16 (76.2%)	0.79
No	94 (35.3%)	172 (64.7%)		149 (56.4%)	115 (43.6%)		230 (86.5%)	36 (13.5%)		57 (21.4%)	209 (78.6%)	
*Mental illness*												
Yes	21 (37.5%)	35 (62.5%)	0.93	35 (62.5%)	21 (37.5%)	0.59	49 (87.5%)	7 (12.5%)	0.67	17 (30.4%)	39 (69.6%)	0.08
No	88 (38.1%)	143 (61.9%)		134 (58.5%)	95 (41.5%)		197 (85.3%)	34 (14.7%)		45 (19.5%)	186 (80.5%)	
*Breathing disorder*												
Yes	31 (57.4%)	23 (42.6%)	**0.001**	46 (85.2%)	8 (14.81%)	**<0.0001**	46 (85.2%)	8 (14.8%)	0.90	15 (27.8%)	39 (72.2%)	0.22
No	78 (33.5%)	155 (66.5%)		123 (53.3%)	108 (46.8%)		200 (85.8%)	33 (14.2%)		47 (20.2%)	186 (79.8%)	
*Other chronic condition*												
Yes	41 (43.6%)	53 (56.4%)	0.17	68 (73.9%)	24 (26.1%)	**0.0005**	82 (87.2%)	12 (12.8%)	0.61	21 (22.3%)	73 (77.7%)	0.83
No	68 (35.2%)	125 (64.8%)		105 (52.3%)	92 (47.7%)		164 (85.0%)	29 (15.0%)		41 (21.2%)	152 (78.8%)	

Bold values denote statistically significant *p* values.

### Emotional factors: anxiety and distress (IES) across sample characteristics

Table 4 shows the association between respondent characteristics, anxiety, and distress (IES). Notably, minority respondents had mean scores significantly higher on anxiety and all three sub-scores of the IES compared with White/non-Hispanics (*p* = 0.01, <0.0001, 0.0018, 0.0014, respectively). Age was also a notable respondent characteristic, with respondents 65 or older reporting lower mean scores on anxiety and all sub-scales of the IES compared with younger participants. Respondents reporting mental illness also had mean scores significantly higher on anxiety and all sub-scores of the IES compared with respondents not reporting mental illness as a comorbidity. These results reveal significant variability in respondents’ levels of anxiety and distress associated with minority status, age, and reported mental illness. Minority respondents reported significantly higher levels of anxiety on all dimensions of the IES compared with White/non-Hispanics.

**Table 4:** Anxiety, and distress (IES) across sample characteristics.

	Anxiety scale score		Impact of events scale score					
			Intrusion sub-score		Avoidance sub-score		Hyperarousal sub-score	
	Mean (SD)	*p*-value	Mean (SD)	*p*-value	Mean (SD)	*p*-value	Mean (SD)	*p*-value
*Global score*	2.34 (0.52)	NA	2.26 (0.83)	NA	2.00 (0.64)	NA	2.02 (0.85)	NA
White, non-Hispanic	2.28 (0.49)	**0.01**	2.1 (0.76)	**<0.0001**	1.19 (0.60)	**0.002**	1.89 (0.78)	**0.001**
Minority	2.45 (0.52)		2.6 (0.88)		2.19 (0.72)		2.28 (0.96)	
*Age*								
18–45	2.42 (0.58)	**<0.0001**	2.44 (0.91)	**<0.0001**	2.18 (0.75)	**0.002**	2.34 (0.89)	**<0.0001**
46–64	2.45 (0.47)		2.49 (0.84)		2.09 (0.67)		2.25 (0.93)	
65+	2.21 (0.49)		1.97 (0.68)		1.83 (0.52)		1.67 (0.60)	
*Gender*								
Male	2.18 (0.50)	**0.01**	1.86 (0.75)	**<0.0001**	1.89 (0.59)	0.20	1.74 (0.81)	**0.002**
Female	2.37 (0.50)		2.36 (0.82)		2.02 (0.66)		2.09 (0.86)	
*Employment*								
Yes	2.35 (0.51)	0.50	2.33 (0.85)	0.19	1.98 (0.66)	0.42	2.08 (0.87)	0.22
No	2.31 (0.50)		2.21 (0.82)		2.02 (0.65)		1.96 (0.85)	
*Lost job*								
Yes	2.37 (0.51)	0.68	2.39 (0.77)	0.24	2.02 (0.66)	0.91	2.21 (0.78)	0.06
No	2.33 (0.51)		2.27 (0.85)		2.00 (0.68)		2.01 (0.88)	
*Cancer*								
Yes	2.26 (0.47)	0.07	2.12 (0.78)	**0.04**	1.94 (0.59)	0.38	1.89 (0.77)	0.11
No	2.34 (0.53)		2.33 (0.85)		2.03 (0.67)		2.08 (0.88)	
*Diabetes*								
Yes	2.40 (0.52)	0.62	2.22 (0.91)	0.69	2.19 (0.73)	0.14	1.89 (0.94)	0.23
No	2.34 (0.52)		2.27 (0.82)		1.98 (0.64)		2.03 (0.85)	
*Mental illness*								
Yes	2.57 (0.56)	**0.0001**	2.71 (0.95)	**<0.0001**	2.25 (0.78)	**0.005**	2.63 (0.91)	**<0.0001**
No	2.28 (0.49)		2.15 (0.76)		1.94 (0.59)		1.87 (0.77)	
*Breathing disorder*								
Yes	2.32 (0.53)	0.75	2.45 (1.02)	0.25	2.17 (0.86)	0.23	2.28 (1.03)	0.07
No	2.35 (0.51)		2.22 (0.77)		1.96 (0.58)		1.96 (0.79)	
*Other chronic condition*								
Yes	2.43 (0.56)	**0.04**	2.36 (0.86)	0.2756	2.13 (0.71)	**0.03**	2.17 (0.88)	**0.03**
No	2.30 (0.49)		2.22 (0.81)		1.94 (0.60)		1.95 (0.83)	

Bold values denote statistically significant *p* values.

### Emotional factors: intolerance of uncertainty across sample characteristics

As shown in Table 5, minority respondents had significantly higher mean scores on the 5-point inhibitory factor of the IU, which is the behaviorally focused dimension of the IU and measures avoidance and paralysis in the face of uncertainty. There were no significant differences in the 7-point dimension, which measures prospective anxiety, desire for predictability, and active engagement in seeking information to increase certainty. Respondents under the age of 65 had significantly higher mean IU scores on both the 12-point scale and the 5-point dimension compared to respondents 65 and older. Respondents reporting mental health as a comorbidity had higher significant mean scores on the 12-point IU scale and on both the 5-point and 7-point dimensions of the IU compared to respondents not reporting a mental health comorbidity.

**Table 5:** Intolerance of uncertainty across sample characteristics.

	Intolerance for uncertainty scale (12 point)		Intolerance for uncertainty scale (5 point—inhibit)		Intolerance for uncertainty scale (7 point—perspective)	
	Mean (SD)	*p*-value	Mean (SD)	*p-*value	Mean (SD)	*p*-value
*Global score*	27.91 (9.72)	NA	9.58 (4.49)	NA	18.32 (6.05)	NA
*Race*						
White, non-Hispanic	27.01 (8.89)	0.08	8.86 (3.99)	**0.0004**	18.15 (5.74)	0.71
Minority	29.84 (11.04)		11.11 (5.06)		18.73 (6.68)	
*Age*						
18–45	31.17 (10.74)	**0.003**	11.44 (5.02)	**0.0002**	19.73 (6.51)	0.032
46–64	29.13 (10.82)		10.04 (4.67)		19.10 (6.97)	
65+	25.39 (7.41)		8.37 (3.68)		17.03 (4.60)	
*Gender*						
Male	27.27 (9.68)	0.62	8.98 (4.49)	0.15	18.29 (6.02)	0.99
Female	28.05 (9.75)		9.72 (4.49)		18.33 (6.07)	
*Employed*						
Yes	28.22 (10.17)	0.23	9.76 (4.50)	0.46	18.46 (6.48)	0.66
No	27.37 (8.93)		9.30 (4.31)		18.07 (5.45)	
*Lost job*						
Yes	28.60 (10.45)	0.69	10.58 (4.95)	0.09	18.02 (6.13)	0.66
No	27.71 (9.76)		9.29 (4.39)		18.42 (6.15)	
*Cancer*						
Yes	27.31 (8.85)	0.89	9.16 (4.07)	0.54	18.15 (5.63)	0.94
No	28.19 (10.11)		9.78 (4.67)		18.40 (6.25)	
*Diabetes*						
Yes	26.33 (10.52)	0.26	9.29 (5.28)	0.43	17.05 (5.62)	0.21
No	28.04 (9.66)		9.61 (4.43)		18.43 (6.08)	
*Mental illness*						
Yes	32.22 (9.76)	**<0.0001**	11.84 (4.48)	**<0.0001**	20.38 (6.08)	**0.004**
No	26.83 (9.43)		9.02 (4.32)		17.81 (5.95)	
*Breathing disorder*						
Yes	27.93 (10.35)	0.88	9.80 (5.10)	0.93	18.13 (6.10)	0.79
No	27.90 (9.59)		9.53 (4.34)		18.37 (6.05)	
*Other chronic condition*						
Yes	29.61 (9.99)	**0.04**	10.32 (4.63)	**0.03**	19.29 (6.09)	0.06
No	27.04 (9.50)		9.21 (4.38)		17.83 (5.99)	

Bold values denote statistically significant *p* values.

### Associations between emotional factors (anxiety, IU, IES) and cognitive factors (threat and coping appraisals)

A multivariate logistic regression model was conducted to assess whether age, White/non-Hispanic vs. minority, gender, anxiety, distress (IES), and IU significantly predicted perceptions of threat and coping appraisals. As shown in Table 6, age, gender, and minority status were significantly associated with perceptions of severity. Minority status was significantly associated with perceived severity and with self-efficacy beliefs. Age was significantly associated with perceived risk and severity, both components of threat appraisals. None of the emotional level factors, IU, anxiety, and IES, were significantly associated with threat and coping appraisal measures.

**Table 6:** Multivariable models examining patient characteristics, threat, and coping appraisals.

Intercept	Reference		Model 1: COVID-19 would be a serious illness for me.		Model 2: My chances of getting infected with Coronavirus COVID-19 are greater than other people		Model 3: I have little control over whether I will get COVID-19.		Model 4: If I don’t take any preventive actions, then I am likely to get Coronavirus COVID-19.	
		DF	Odds Ratio (95% CI)	*p*-value	Odds ratio (95% CI)	*p*-value	Odds Ratio (95% CI)	*p*-value	Odds ratio (95% CI)	*p*-value
Age	Unit in Year	1	1.071 (1.046, 1.098)	**<0.0001**	1.022 (1.001, 1.044)	**0.04**	0.993 (0.969, 1.018)	0.59	1.001 (0.973, 1.03)	0.96
Gender	1 (Male) vs. 2 (Female)	1	2.588 (1.163, 5.76)	**0.02**	1.936 (0.989, 3.787)	0.05	1.584 (0.677, 3.709)	0.29	0.626 (0.268, 1.46)	0.28
Race	1 (All minorities) vs. 0 (White NH)	1	2.477 (1.278, 4.801)	**0.007**	1.699 (0.938, 3.076)	0.08	2.468 (1.239, 4.916)	**0.010**	2.285 (0.853, 6.119)	0.10
Intolerance to uncertainty	Unit in Intolerance of Uncertainty scale (12 item)	1	1.003 (0.931, 1.08)	0.93	1.033 (0.964, 1.108)	0.35	0.988 (0.91, 1.072)	0.77	0.991 (0.893, 1.099)	0.86
Anxiety	Unit in Anxiety scale	1	1.623 (0.758, 3.476)	0.21	0.764 (0.382, 1.53)	0.44	1.485 (0.632, 3.487)	0.36	1.574 (0.568, 4.36)	0.38
Intrusion	Unit in Intolerance of Uncertainty scale intrusion sub-scale mean	1	1.433 (0.772, 2.659)	0.25	1.703 (0.949, 3.057)	0.07	0.912 (0.464, 1.793)	0.79	1.026 (0.429, 2.451)	0.95
Avoidance	Unit in Intolerance of Uncertainty scale avoidance sub-scale mean	1	1.26 (0.755, 2.104)	0.38	0.855 (0.531, 1.377)	0.51	1.014 (0.583, 1.763)	0.96	0.997 (0.497, 2.002)	0.99
Hyperarousal	Unit in Intolerance of Uncertainty scale hyperarousal sub-scale mean	1	0.937 (0.502, 1.749)	0.84	0.912 (0.506, 1.644)	0.76	1.441 (0.743, 2.796)	0.28	1.182 (0.477, 2.929)	0.72

Bold values denote statistically significant *p* values.

To explore the relationships between the cognitive factors and emotional level factors, we subsequently attempted to explore the mediating effects of threat and coping appraisals and IE on IU and anxiety using structural modeling. A full overall predictive model with threat and coping appraisals and IE pathways were fitted and compared with four alternative models featuring (1) no mediators, (2) only threat appraisals as mediating variables, (3) only coping appraisals as mediators, and (4) only impact of events as mediating variables.

As shown in Table 7, positive correlations existed between IU and the coping appraisals ‘response efficacy’ and ‘self-efficacy,’ which were stronger than the correlations with threat appraisals. Weak correlations were also found between coping appraisals and anxiety. All IE dimensions, ‘intrusion’, ‘avoidance’, and ‘hyperarousal’ were strongly correlated with IU and anxiety. Coping appraisals were correlated with each other; this was the same case with threat appraisals and all IE dimensions. IU itself was positively correlated with anxiety.

**Table 7:** Spearman correlation among IU, threat and coping appraisals, anxiety, and IE.

	1.	2.	3.	4.	5.	6.	7.	8.
1. Intolerance of uncertainty	–							
2. Perceived risk	0.066 (0.276)	–						
3. Perceived severity	0.032 (0.593)	**0.364 (<0.001)**	–					
4. Response efficacy	**0.155 (0.010)**	**0.196 (0.001)**	**0.149 (0.013)**	–				
5. Self-efficacy	**0.135 (0.025)**	**0.307 (<0.001)**	**0.154 (0.010)**	**0.139 (0.021)**	–			
6. Anxiety	**0.394 (<0.001)**	0.062 (0.308)	**0.149 (0.013)**	**0.223 (<0.001)**	**0.179 (0.003)**	–		
7. Intrusion	**0.442 (<0.001)**	0.095 (0.117)	0.075 (0.215)	**0.120 (0.046)**	**0.126 (0.036)**	**0.660 (<0.001)**	–	
8. Avoidance	**0.300 (<0.001)**	−0.006 (0.916)	0.059 (0.329)	0.077 (0.205)	0.117 (0.052)	**0.331 (<0.001)**	**0.413 (<0.001)**	–
9. Hyperarousal	**0.511 (<0.001)**	0.036 (0.557)	0.015 (0.801)	0.098 (0.104)	0.114 (0.059)	**0.633 (<0.001)**	**0.787 (<0.001)**	**0.465 (<0.001)**

Bold values denote statistically significant *p* values.

None of the three mediator models improved over the no-mediator model of IU predicting anxiety (Table 8). While the mediator model featuring coping appraisals demonstrated a better fit over the threat appraisals, the model still performed notably worse than the direct effect model. This was also seen in the IE factors model, which demonstrated a comparative fit index (CFI) better than the full predictive model but a worse fit than with no mediators. Thus, the predicted model with mediators was not a good fit, and any mediating model was less than one without mediators. We also found no difference between minorities and non-minorities (White non-Hispanics) and the pathways from IU to anxiety. These findings suggest IU predicted anxiety levels and that cognitive factors (threat and coping appraisals) failed to mediate.

**Table 8:** Comparative fit indices for structural models.

Model	*X*^*2*^	df	*p*	NNFI	CFI	RMSEA	RMSEA CI
Predictive (all mediators)	341.681	28	<0.001	0	0.075	0.194	(0.177, 0.212)
Model 1—No mediators	55.091	1	<0.001	1	1	0	(0, 0)
Model 2—threat	74.992	3	<0.001	0.059	0.059	0.287	(0.231, 0.346)
Model 3—coping	21.845	3	<0.001	0.865	0.865	0.056	(0, 0.127)
Model 4—IE	615.459	10	<0.001	0.321	0.525	0.386	(0.349, 0.424)

## Discussion and conclusions

By conducting this survey early in the pandemic, our study evaluated cognitive and emotional factors among a racially/ethnically diverse group of NYC residents during the height of the COVID-19 pandemic. The survey explored the influence of socio-demographics and comorbidities on cognitive and emotional level factors, healthcare utilization, and initiation of preventive behaviors when the emotional and cognitive impact of the pandemic was high on the population level. During this critical and unprecedented point in the pandemic, NYC was mainly in a lock-down state, with 90% of survey respondents reporting “staying home unless doing something absolutely essential”. All participants reported at least one comorbidity in this diverse high-risk sample of adults. Almost all participants reported changing at least one health behavior over the past 4 days due to COVID-19. Our data show how healthcare utilization was interrupted early in the pandemic, with 51% of respondents not being able to receive care through their provider as planned and only 17% reporting the ability to receive healthcare through telemedicine. Previous studies have reported disruptions in healthcare during different periods of the pandemic. In June 2020, a CDC report used a web-based survey to show that around 40.9% of U.S. adults had forgone medical care (Czeisler et al., 2020). In September 2020, using data from the Coronavirus Tracking Survey, the Urban Institute reported that 36% of U.S. nonelderly adults had delayed healthcare utilization (Gonzalez et al., 2021). Our finding of 51% is higher than the previously reported prevalence and is particularly concerning given the comorbidities reported by our respondents. An explanation could be that participants in our survey were from a particularly disadvantaged community, with fewer resources to support healthcare access during the pandemic, including the computer access and literacy needed to take advantage of telemedicine-based services.

Few studies have examined associations between minority status and cognitive and emotional factors during a pandemic. One study during the early onset of COVID-19 in the U.S. examined individuals’ perceived susceptibility and found that African American adults felt least prepared to deal with the pandemic and reported less perceived susceptibility to the virus (Bailey et al., 2020). Another study also found that African Americans were significantly less likely to perceive they were at risk of COVID-19 infection compared to their White counterparts, which mirrors similar perceptions during the H5N1 and H1N1 outbreaks (Scarinci et al., 2021). Our findings revealed that minority respondents rated their likelihood of getting COVID-19 if they didn’t take preventive actions significantly higher compared to White/non-Hispanic respondents (response efficacy). They were also significantly more likely to report having little control over whether they will get COVID-19 compared with White/non-Hispanic respondents (self-efficacy). Notably, minority respondents had mean scores significantly higher on anxiety and all three sub-scores of the IES measuring dimensions of distress than White/non-Hispanics. Minority respondents also had significantly higher mean scores on the 5-point inhibitory factor of the IU compared with White/non-Hispanic respondents, which is the behaviorally focused dimension of the IU and measures avoidance and paralysis in the face of uncertainty. Together, these data indicate that threats related to the pandemic have implications for sharp disparities in coping and psychological distress disproportionately experienced by minority groups.

Like our study, Sadeh et al. examined the relationships between distress and IU in minority groups, comparing Black and White adults drawn from socioeconomically disadvantaged neighborhoods. Findings revealed that mental health consequences of scoring high on IU appeared to be more severe for Black than White adults, providing indirect evidence that race-related uncertainty may impact the well-being of Black adults (Sadeh and Bounoua, 2022). The authors postulated that structural racism and discrimination might cause elevated uncertainty in daily life for Black Americans, which in turn has negative mental health consequences for Black adults high on IU (To et al., 2020; Sue et al., 2008). Interestingly, they found that race interacted with IU among participants who participated before the pandemic started, but it did not moderate IU associations with mental health for individuals with pandemic exposure, as both Black and White adults showed positive associations between IU and mental health post-pandemic (Sadeh and Bounoua, 2022). Our study revealed significantly higher levels of IU, anxiety, and all three sub-scales of IES measuring dimensions of distress in the minority respondents compared with White/non-Hispanic respondents. However, we did not measure differences pre-pandemic, and compared with White counterparts, the heightened IU, stress, and anxiety for minority/non-White respondents continued during the pandemic. Whether this heightened IU is due to pre-pandemic elevated uncertainty in the daily life of minority populations or if there are other reasons for differential responses to pandemic uncertainty is an area for continued investigation.

In the introduction, we noted that few attempts had been made to converge cognitive behavioral antecedents with emotional factors. We also noted that explanatory variables from behavioral theories to predict how people and different population subgroups respond to emerging infectious diseases seldom focus on IU. While this is a complex mix of factors to explain the pandemic response, COVID-19 response variations among population subgroups require a complex explanatory context. Chowkwanyun and Reed eloquently address the importance of explanatory context in their perspective on racial health disparities and COVID-19 (Chowkwanyun and Reed, 2020). The authors caution that citing racial disparities to COVID-19 without explanatory context can potentially perpetuate harmful myths and misunderstandings that undermine the goal of eliminating health inequities. To mitigate myths and specifically to avoid inaccurate assumptions grounded in racial biology (Chowkwanyun and Reed, 2020), COVID-19 disparities should be discussed in the context of socio-demographics, chronic stress, or place-based (e.g., community) risk. Our contextualization of factors that include cognitive and emotional aspects can help to explain why racial minority populations are affected disproportionally and may also provide guidance to developers of future pandemic response interventions.

Although our results are preliminary and replication studies will be needed, our findings suggest the need to develop culturally responsive messaging and interventions during pandemics. For example, exploring the effect of race-related differences in exposure to uncertainty as a key source of anxiety and distress in minority populations may lead to programs that pay high attention to the mental health problems evoked by intolerance of uncertainty. This type of intervention will likely require the expertise of many disciplines, including mental health experts, to address the emotional and behavioral responses and communication experts to address the exasperation of uncertainty, particularly among the most vulnerable populations. Because few studies have reported differences by race and ethnicity during pandemic exposure, further research on factors that may influence heightened IU, stress, and anxiety among minority populations is needed.

Despite many novel findings, there are several limitations in our study. First, our survey was administered using a pop-up on a community health portal, thus limiting access to participants with access to the Internet. Furthermore, we utilized all self-report measures with limitations such as potential social desirability bias, exaggeration, or underreporting. The study’s cross-sectional design makes it impossible to infer the exact causal relationship between variables. Further longitudinal work is needed to examine the interplay of cognitive factors, IU, and heightened stress and anxiety over time to shed light on the causal mechanisms driving these differential effects across racial and ethnic groups. Despite these limitations, by conducting this survey early in the pandemic, our study uniquely evaluated cognitive and emotional factors among a racially/ethnically diverse group of NYC residents during the height of the COVID-19 pandemic. Findings allowed us to identify disparate responses among minority respondents, with implications for exploring how to tailor future pandemic response interventions. More extensive studies are warranted to investigate further the underlying factors associated with disparities illuminated during the COVID-19 pandemic and the mechanisms to optimize future pandemic response interventions.

## Data Availability

The data used in the current study are available on request from the corresponding author. The data are not publicly available due to privacy or ethical restrictions.
